# Sunitinib induces macrophage dysfunction and impaired tissue regeneration through suppression of PPARγ

**DOI:** 10.3389/fimmu.2026.1900449

**Published:** 2026-08-14

**Authors:** Zhouchen Zhang, Qiao Qiao, Jiannan Li, Qingxiang Li, Yuanning Yang, Ying Zhou, Hongyuan Huang, Dongyan Chen, Yifei Wang, Yuxing Guo

**Affiliations:** 1 Department of Oral and Maxillofacial Surgery, Peking University School and Hospital of Stomatology, Beijing, China; 2 National Clinical Research Center for Oral Diseases, Peking University School and Hospital of Stomatology, Beijing, China; 3 National Engineering Laboratory for Digital and Material Technology of Stomatology, Peking University School and Hospital of Stomatology, Beijing, China; 4 Beijing Key Laboratory of Digital Stomatology, Peking University School and Hospital of Stomatology, Beijing, China; 5 General Dentistry, Peking University School and Hospital of Stomatology, Beijing, China; 6 Department of Histology and Embryology, School of Medicine, Nankai University, Tianjin, China

**Keywords:** macrophage, PPARγ, rosiglitazone, sunitinib, tissue regeneration

## Abstract

**Background:**

Sunitinib is a widely used multi-target tyrosine kinase inhibitor associated with side effects that may impair tissue repair and regeneration, potentially contributing to the onset of medication-related osteonecrosis of the jaw. Macrophages play a central role in tissue homeostasis and regeneration, and their dysfunction may be a key factor in this pathological process.

**Methods:**

In this study, a zebrafish tail fin injury regeneration model was used to investigate the effects of sunitinib on macrophage function and tissue regeneration. Transcriptomic analysis, immunofluorescence staining, RT-qPCR, and Seahorse mitochondrial stress assays were performed to elucidate the underlying mechanisms.

**Results:**

Sunitinib treatment significantly impaired macrophage migration and tissue regeneration in a concentration-dependent manner. Transcriptomic analysis revealed that sunitinib markedly suppressed the PPARγ signaling pathway, reparative gene programs, and mitochondrial metabolism. The density of PPARγ+ macrophages at the wound site was significantly reduced following sunitinib exposure. Treatment with rosiglitazone, a PPARγ agonist, effectively rescued the regeneration defects induced by sunitinib, restored macrophage infiltration, and recovered oxidative phosphorylation. Notably, these rescuing effects were abolished upon macrophage depletion.

**Discussion:**

Our results demonstrate that sunitinib impairs tissue regeneration by suppressing PPARγ signaling and oxidative phosphorylation in macrophages. Targeting the PPARγ pathway represents a promising therapeutic strategy to reverse sunitinib-associated deficits in tissue repair.

## 1 Introduction

Sunitinib is a multi-target receptor tyrosine kinase inhibitor widely used in the clinical treatment of various malignancies, including renal cell carcinoma and gastrointestinal stromal tumors (1, 2). It exerts its antitumor effects primarily by potently inhibiting key signaling pathways such as vascular endothelial growth factor receptor (VEGFR), platelet-derived growth factor receptor (PDGFR), and stem cell factor receptor (cKIT), thereby significantly suppressing tumor angiogenesis and cell proliferation (3). However, alongside its therapeutic benefits, sunitinib is associated with a range of adverse reactions, including hypertension, arterial thromboembolic events (ATE), proteinuria or renal dysfunction, bleeding, gastrointestinal perforation, cardiotoxicity, hypothyroidism, and dermatological toxicities (4, 5). Early reports have indicated that sunitinib may impair the tissue injury repair process, potentially leading to the non-healing of previous or subsequent surgical wounds, although the underlying mechanisms remain unclear (4). Therefore, fundamental pharmacological research on this issue is highly necessary.

Tissue repair is a dynamic process involving multiple cells and stages, which can be broadly divided into four stages: hemostasis, inflammation, proliferation, and remodeling (6). The inflammatory phase initiates the tissue’s response to injury, recruiting immune cells such as neutrophils and macrophages to clear pathogens and necrotic debris. While the initiation of inflammation is essential for repair, its timely resolution is critical for creating appropriate conditions for subsequent regeneration. Previous studies have shown that excessive inflammatory responses can be detrimental to recovery, as high levels of inflammatory cytokines can inhibit proliferation and migration of fibroblasts, endothelial cells, and stem cells, characterized by reduced angiogenesis and insufficient extracellular matrix deposition, ultimately delaying tissue regeneration (7, 8).

Macrophages are of great importance in tissue repair and regeneration. They not only clear apoptotic cells and debris but also regulate inflammation and promote tissue reconstruction by secreting various cytokines and growth factors (9, 10). There is research indicating that functional or numerical deficiencies in macrophages can directly lead to delayed or failed regeneration (11, 12). Consequently, we hypothesize that sunitinib compromises the regenerative microenvironment by impairing macrophage function, thereby disrupting wound healing and ultimately leading to severe complications such as medication-related osteonecrosis of the jaw (MRONJ).

This study employed a zebrafish tail fin injury regeneration model and demonstrated the effect of sunitinib on macrophages at the wound site, and revealed its inhibitory effect on the peroxisme proliferator-activated receptor gamma (PPARγ) signaling pathway and the oxidative phosphorylation in macrophages. These findings provide a potential therapeutic target for sunitinib-induced impairment of tissue healing.

## 2 Materials and methods

### 2.1 Zebrafish husbandry and embryo handling

AB strain zebrafish (Danio rerio) and Tg(MPEG-GFP) transgenic zebrafish were used in this study. All fish were maintained at 28.5 °C under a standardized light cycle of 14 hours light/10 hours dark. Embryos were collected through natural spawning and cultured in E3 embryo medium. Experiments were conducted using embryos at specific developmental stages, and all procedures adhered to internationally recognized animal welfare guidelines.

### 2.2 Drug treatment

Sunitinib (Su, S7781, Selleck, USA) and Rosiglitazone (Rosi, S2556, Selleck, USA) were dissolved in DMSO to prepare stock solutions, which were stored at -20 °C. The stock solutions were diluted in embryo medium (E3) to obtain working solutions with the final DMSO concentration maintained at 0.1%. Zebrafish at 2 days post-fertilization (2 dpf) were immersed in working solutions until 3 dpf. Following the onset of tail fin injury, the work solution was replaced daily until the designated endpoints. The concentration of sunitinib was 8 μM, which is above the concentration range known to affect macrophage, below the estimated human-equivalent concentration and the toxicity threshold (13, 14).

### 2.3 Macrophage-depleted model

To specifically deplete macrophages, antisense morpholino oligonucleotide (MO)-mediated gene knockdown was performed. An MO sequence targeting the translation start site of irf8—a key gene in macrophage development—was designed: 5’-AATGTTTCGCTTACTTTGAAAATGG-3’ (Gene Tools, USA). Zebrafish embryos at the 1- to 2-cell stage were micro-injected with 1–2 nL of the MO solution, followed by continued culture. Knockdown efficiency was subsequently verified using macrophage-specific fluorescent labeling.

### 2.4 Tail fin amputation and regeneration model

Zebrafish larvae at 3 days post-fertilization (3 dpf) were anesthetized with Tricaine. Under a microscope, tail fin amputation was performed using a sterile surgical blade by transecting the fin perpendicular to the body axis at the level of the posterior end of the notochord. Immediately after amputation, zebrafish larvae were transferred into fresh drug solution or system water to recover and initiate the regeneration process (15). The regeneration process was monitored and recorded under a microscope.

### 2.5 Measurement of tail fin regrowth length

AB strain zebrafish larvae at 3 days post-fertilization (3 dpf) were used. At 6 hours post-amputation, tail fin regeneration was examined under an microscope. The regrowth length was measured from the distal end of the notochord at the level of the spinal column to the edge of the regenerating tissue.

### 2.6 Macrophage observation

Tg(MPEG-GFP) transgenic zebrafish larvae at 3 days post-fertilization were used. Macrophage numbers were observed under an fluorescence microscope at 6 hours post-amputation. The region of interest for quantification included a rectangular area extending from the amputation plane to the distal edge of the regenerating tissue. To evaluate macrophage migratory activity, macrophage numbers were also examined at 1, 2, 3, 4, 5, and 6 hours post-amputation (1, 2, 3, 4, 5, 6 hpa) under the fluorescence microscope.

### 2.7 Cell proliferation assay (BrdU staining)

To assess cell proliferation ability, zebrafish larvae were incubated in the corresponding working solution containing 5 mM 5-bromo-2’-deoxyuridine (BrdU, B5002, Sigma-Aldrich, USA) for 12 hours starting at 12 hours post-amputation. Subsequently, the larvae were fixed in 4% paraformaldehyde (PFA) for 2 hours, washed three times for 5 minutes each in PBS, and then subjected to antigen retrieval by incubation in 2 N HCl at 37 °C for 30 minutes, followed by another three 5-minute PBS washes. After permeabilization with 0.5% Triton X-100 (A16046.AP, BBI life sciences, China) for 40 minutes, samples were blocked with 5% goat serum for 2 hours at room temperature. The samples were then incubated with an anti-BrdU antibody (1:200 dilution, 61274, Proteintech, USA) overnight at 4 °C. After washing three times in PBS (5 minutes each), a Cy3-conjugated secondary antibody (1:200 dilution, SA00009-1, Proteintech, USA) was applied and incubated at 37 °C for 1 hour. Following a final series of three 5-minute PBS washes, proliferating cells were visualized under a fluorescence microscope.

### 2.8 Detection of apoptotic cells (acridine orange staining)

To detect apoptotic cells *in vivo*, a 1000× stock solution of acridine orange (AO, A1303, Gentihold, China) was prepared by dissolving 20 mg in E3 medium (10mL), which was then diluted to obtain the working solution. Zebrafish larvae were incubated in 1× AO working solution for 30 min in the dark, followed by three 5-minute washes with E3 medium. Apoptotic cells were immediately visualized and imaged under a fluorescence microscope.

### 2.9 Detection of PPARγ expression in macrophage

Three dpf MPEG-GFP transgenic zebrafish larvae were fixed in 4% paraformaldehyde for 2 h at room temperature at 6 h post-amputation (hpa) following caudal fin resection. After washing with PBS containing 0.1% Triton X-100, samples underwent antigen retrieval in 0.1 M Tris-HCl (pH 9.0) at 70 °C for 15 min, followed by permeabilization with pre-cooled acetone (-20 °C) for 10 min. Residual acetone was removed by PBS. Samples were then blocked with 10% goat serum for 30 min at room temperature, and incubated overnight at 4 °C with anti-GFP (1:200, AE012, Abclonal, China) and rabbit anti-PPARγ (1:200) primary antibodies. After PBS washes, samples were incubated with Alexa Fluor 488-conjugated goat anti-mouse IgG (1:200, A0270SP, Abclonal, China) and Alexa Fluor 594-conjugated goat anti-rabbit IgG (1:200) secondary antibodies for 1.5 h at room temperature in the dark. Following PBS washes, samples were imaged under a fluorescence microscope.

### 2.10 Transcriptome sequencing (RNA-seq) and bioinformatics analysis

Tail fin tissue samples were collected from zebrafish larvae treated with sunitinib or DMSO (three biological replicates, each containing >60 larvae). Total RNA was extracted using TRIzol reagent (15596026CN, Invitrogen, USA). After RNA quality control, eukaryotic strand-specific sequencing libraries were constructed. Paired-end 150 bp sequencing was performed on the Illumina NovaSeq 6000 platform. The raw reads were subjected to quality assessment and filtering, aligned to the zebrafish reference genome (GRCz11), and quantified for gene expression. Differential expression analysis was performed using DESeq2 to identify differentially expressed genes (DEGs). Functional enrichment analysis of DEGs was conducted based on Gene Ontology (GO) terms and Kyoto Encyclopedia of Genes and Genomes (KEGG) pathways. The gene set enrichment analysis was performed to analyze macrophage-specific transcriptional programs.

### 2.11 Mitochondrial respiration

The head kidney (the primary reservoir of mature macrophages in adult zebrafish) of Tg(MPEG-GFP) transgenic zebrafish aged 3–6 months were dissected and the single-cell suspension were prepared (16). Then the cell suspension was filtered through a 40 μm cell strainer. Then the macrophage were isolated based on GFP using a BD FACSAria cell sorter. The sorted cells were seeded into culture plates with Leibovitz’s L-15 + 20% FBS+M-CSF (20 ng/mL) for subsequent experiments. Then the Seahorse XF mito stress test (Agilent Technologies, USA) was performed according to the manufacturer’s instructions.

### 2.12 Quantitative real-time PCR

Tail fin tissue samples were collected from zebrafish larvae treated with sunitinib or DMSO (three biological replicates, each containing >60 larvae). Total RNA was extracted using TRIzol reagent. Extracted RNA was reverse-transcribed into cDNA using a reverse transcription kit (CW2141S, CWBIO, China). Amplification reactions were performed on a QuantStudio series real-time PCR instrument using SYBR Green premix, with *β-actin* serving as the control gene. Relative gene expression levels were calculated using the 2^(–ΔΔCt) method. The primers used are listed below. *Il1b*-F, 5’-CTGGAGATGTGGACTTCGCA-3’; *il1b*-R, 5’-TCACGCTCTTGGATGACGTT-3’; *Tnfα*-F, 5’-GCAATCCGCTCAATCTGCAC-3’; *Tnfα*-R, 5’-AGAAGTGCTGTGGTCGTGTC-3’; *il6*-F, 5’-GGCATTTGAAGGGGTCAGGA-3’; *il6*-R, 5’-GCGTTAGACATCTTTCCGTGC-3’; *cxcl8a*-F, 5’-TGAGCTTGAGAGGTCTGGCT-3’; *cxcl8a*-R, 5’-TTTTCCAATGCGTCGGCTTT-3’; *β-actin*-F, 5’-TTCACCACCACAGCCGAAAGA-3’, *β-actin*-R, 5’-TACCGCAAGATTCCATACCCA-3’; *fabp11a*-F, 5’-GGCAAACTTGTGCAGAAACA-3’; *fabp11a*-R, 5’-GAACTGAGCCTGGCATCTTC-3’; *cd36*-F, 5’-TACTTGCTGCCTGTTGATGC-3’; *cd36*-R, 5’-ATTCGTTTTTGACGGTTTCG-3’; *mrclb*-F, 5’-GACAAGAAGGACTGGTACTG-3’; *mrclb*-R, 5’-TATTCACCTCAACTGGAGTC-3’.

### 2.13 Statistical analysis

Image processing and fluorescence intensity analysis were performed using ImageJ software. All experiments were independently repeated at least three times. Data are presented as mean ± SEM. Statistical analyses were conducted using GraphPad Prism 9.0. Comparisons between two groups were performed using Student’s *t*-test, while comparisons among multiple groups were analyzed by one-way or two-way analysis of variance (ANOVA). A *p*-value < 0.05 was considered statistically significant (indicated in figures as *, *p* < 0.05; **, *p< 0.01*;***, *p< 0.001*;****, *p* < 0.0001).

## 3 Results

### 3.1 Sunitinib inhibited wound repair and regeneration

**Figure 1:**
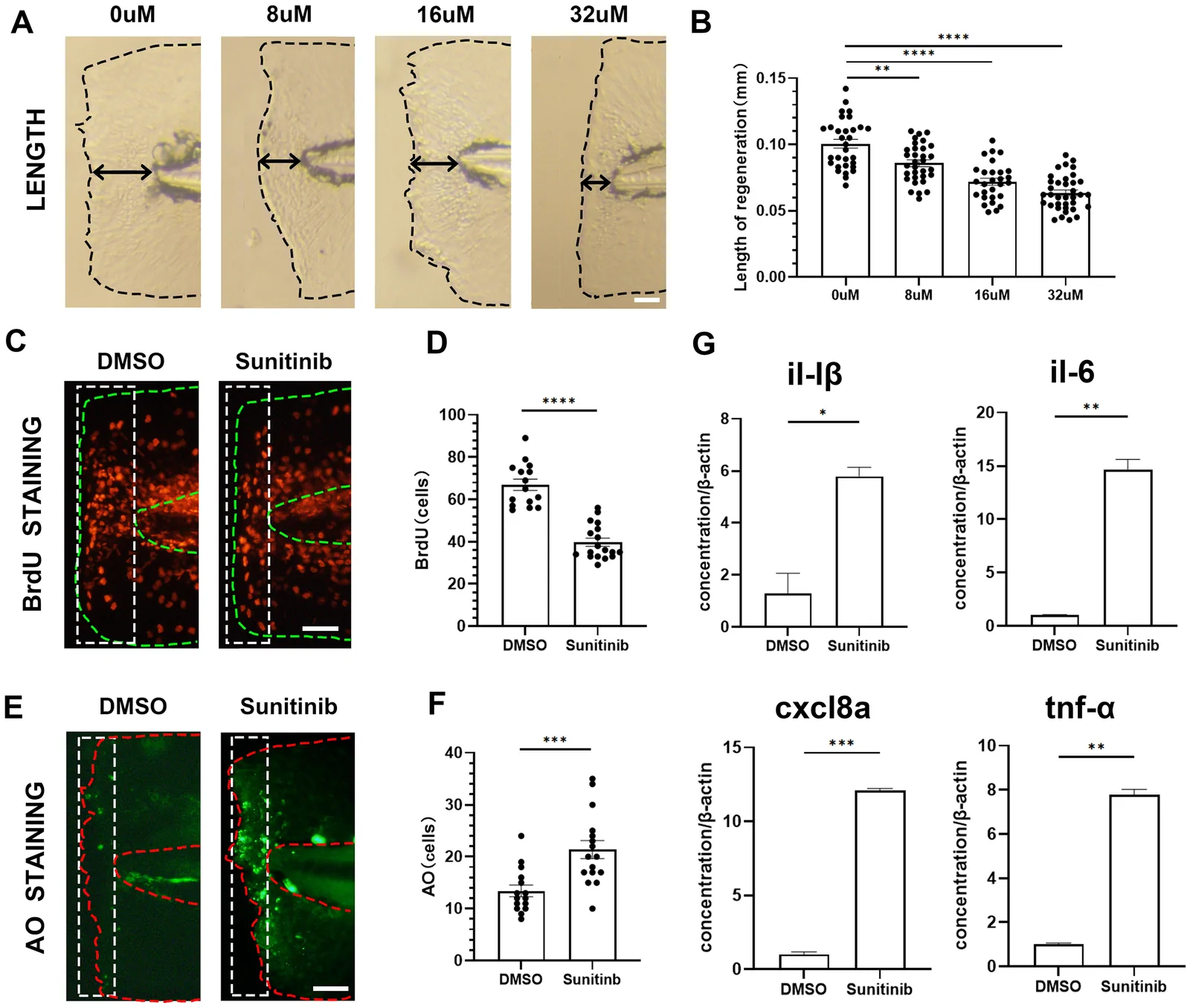
Sunitinib inhibits tissue repair. **(A)** Images of tail fin two days after injury. Black arrow: The distance from the distal end of the notochord at the level of the spinal column to the edge of the regenerating tissue was measured as the regeneration length. Scale bar: 50 μm. **(B)** Quantification of the regeneration length. n=31 in 0 μM group, 32 in 8 μM group, 28 in 16 μM group and 36 in 32 μM group. **(C)** Detection of proliferating cells by fluorescence microscopy one day after tail fin injury. Proliferating cells were labeled with BrdU (orange). Green dashed line: Outline of the zebrafish tail fin and spinal column. White box: Proliferating cells within the quantified region. Scale bar: 50 μm. **(D)** Quantification of the cell count data from **(C)** is shown in **(D)**. n=15 in DMSO group and 18 in Sunitinib group. **(E)** Detection of apoptotic cells by fluorescence microscopy six hours after tail fin injury. Apoptotic cells were labeled with AO (green). Red dashed line: Outline of the zebrafish tail fin and spinal column. White box: Apoptotic cells within the quantified region. Scale bar: 50 μm. **(F)** Quantification of the cell count data from **(E)** is shown in **(F)**. n=15 in DMSO group and 16 in Sunitinib group. **(G)** mRNA expression levels of inflammation-related genes in regenerating zebrafish tail fin tissue. n=2. *p < 0.05, **p < 0.01, ***p < 0.001, and ****p < 0.0001.

To simulate the effect of sunitinib on wound healing, we treated zebrafish with sunitinib and established a tail fin injury model. Two days later, the length of the regenerated tail fin tissue from the distal end to the spine’s end along the longitudinal axis was measured to evaluate the healing outcomes. The results demonstrated that sunitinib administration significantly inhibited tail fin regeneration in a concentration-dependent manner (**Figures 1A, B**). Then the group with 8μM sunitinib was selected for further research. BrdU staining showed that sunitinib treatment reduced the number of proliferating cells within the newly formed tail fin tissue (**Figures 1C, D**), while AO staining revealed increased the number of apoptotic cells in the regenerating tissue (**Figures 1E, F**). Wound healing is a multi-phase process (hemostasis, inflammation, proliferation, remodeling), in which the inflammatory response is the most critical (17). RT-qPCR analysis of the newly formed zebrafish tissue showed a marked upregulation of *il-1*, *il-6*, *cxcl8a*, *and tnf-α*, indicating that sunitinib may inhibit tail fin regeneration by aggravating the local inflammatory response (**Figure 1G**).

### 3.2 Macrophage is necessary for successful tissue regeneration in zebrafish

**Figure 2:**
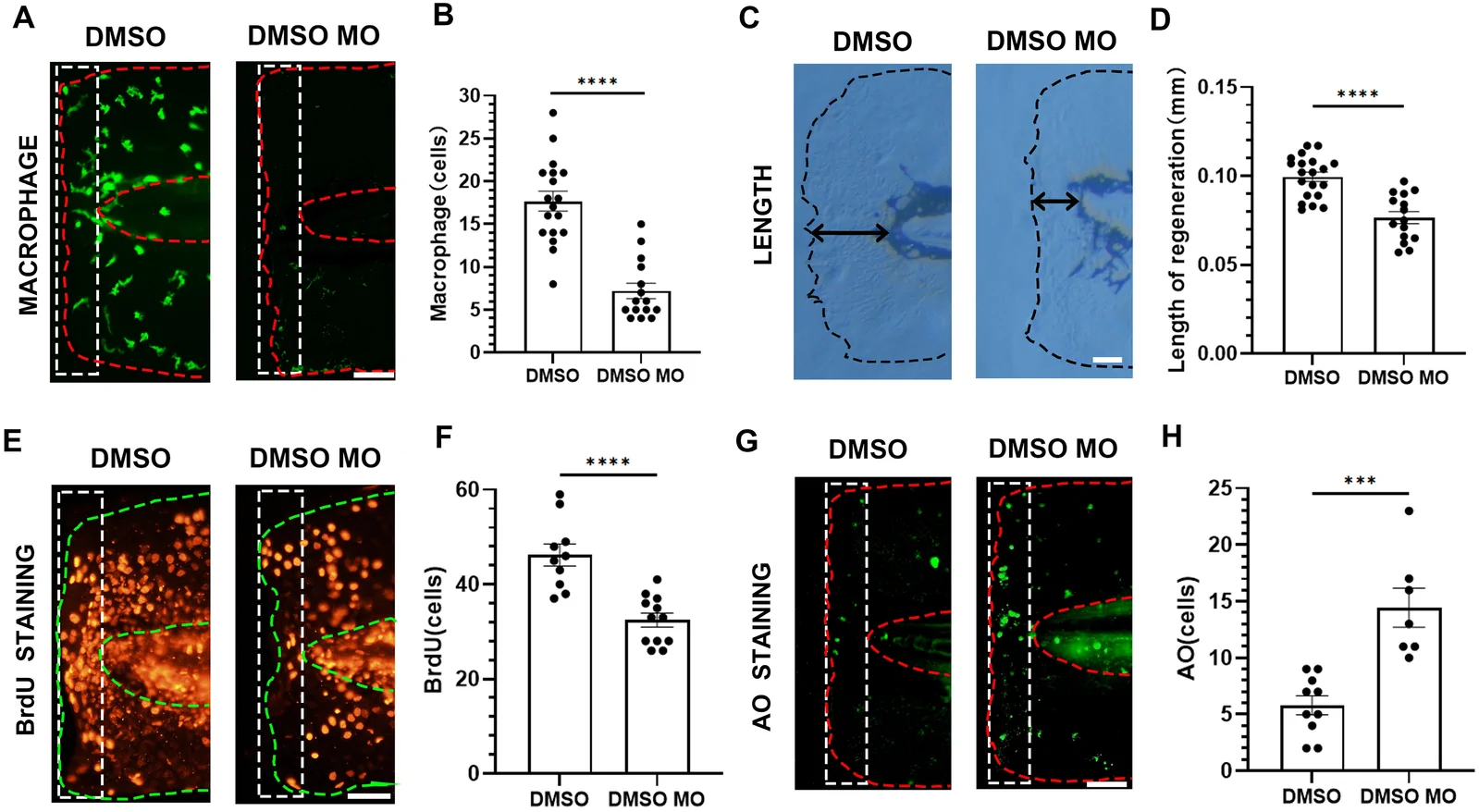
Macrophages are essential for tissue healing in zebrafish. **(A)** Image of tail fin six hours after injury. Macrophages were labeled with MPEG (green). Red dashed line: Outline of the zebrafish tail fin and spinal column. White box: Macrophages within the quantified region. Scale bar: 50 μm. **(B)** Quantification of the cell count data from **(A)** is shown in **(B)**. n=18 in DMSO group and 15 in DMSO MO group. **(C)** Images of tail fin two days after injury. Black arrow: The distance from the distal end of the notochord at the level of the spinal column to the edge of the regenerating tissue was measured as the regeneration length. Scale bar: 50 μm. **(D)** Quantification of the length data from **(C)** is shown in **(D)**. n=20 in DMSO group and 15 in DMSO MO group. **(E)** Detection of proliferating cells by fluorescence microscopy one day after tail fin injury. Proliferating cells were labeled with BrdU (orange). Green dashed line: Outline of the zebrafish tail fin and spinal column. White box: Proliferating cells within the quantified region. Scale bar: 50 μm. **(F)** Quantification of the cell count data from **(E)** is shown in **(F)**. n=10 in DMSO group and 12 in DMSO MO group. **(G)** Detection of apoptotic cells by fluorescence microscopy six hours after tail fin injury. Apoptotic cells were labeled with AO (green). Red dashed line: Outline of the zebrafish tail fin and spinal column. White box: Apoptotic cells within the quantified region. Scale bar: 50 μm. **(H)** Quantification of the data from **(G)** is shown in **(H)**. n=10 in DMSO group and 7 in DMSO MO group. ***p < 0.001, and ****p < 0.0001.

Macrophages orchestrate tissue regeneration by transitioning from pro-inflammatory to pro-repair and pro-resolution states. To verify this role in zebrafish, we used the Tg(MPEG: GFP) transgenic line for macrophage observation. Macrophage depletion was achieved via MO technology, resulting in a significant reduction of macrophages within the tail fin tissue (**Figures 2A, B**). Macrophage depletion significantly reduced the length of regenerated tail fin tissue (**Figures 2C, D**). BrdU staining showed that macrophage depletion decreased the number of proliferating cells within the newly formed tissue (**Figures 2E, F**). AO staining further revealed that macrophage depletion increased the local apoptotic cell count (**Figures 2G, H**). Together, these results suggest that macrophage depletion delays tissue regeneration by suppressing cell proliferation and exacerbating apoptosis.

### 3.3 Sunitinib affects the local infiltration level of macrophages

**Figure 3:**
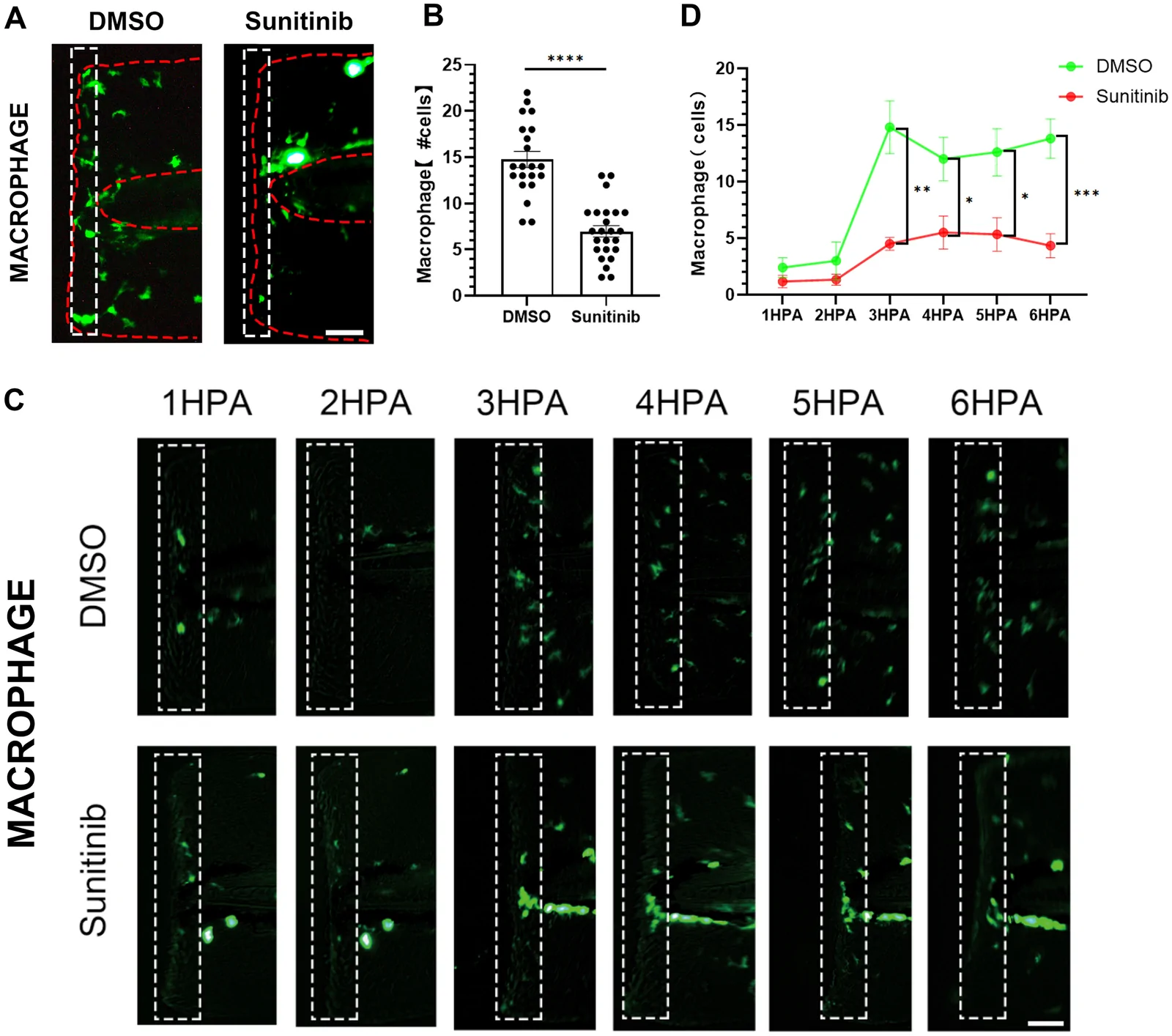
Sunitinib affects macrophage quantity and migration function. **(A)** Image of tail fin six hours after injury. Macrophages were labeled with MPEG (green). Red dashed line: Outline of the zebrafish tail fin and spinal column. White box: Macrophages within the quantified region. Scale bar: 50 μm. **(B)** Quantification of the cell count data from **(A)** is shown in **(B)**. n=21 in DMSO group and 24 in Sunitinib group. **(C)** Images recorded hourly over six hours following tail fin injury. Macrophages were labeled with MPEG (green). White box: Macrophages within the quantified region. Scale bar: 50 μm. **(D)** Quantification of the data from **(C)** is shown in **(D)**. n=5 in DMSO group and 6 in Sunitinib group. *p < 0.05, **p < 0.01, and ***p < 0.001.

Given the substantial impact of macrophage depletion on wound healing, further studies should focus on determining whether sunitinib affects macrophages during tissue regeneration in zebrafish. Consistent with this hypothesis, treatment with sunitinib led to a significant reduction in macrophage numbers within the regenerating tail fin tissue of Tg(MPEG: GFP) transgenic zebrafish (**Figures 3A, B**). We further dynamically monitored the local migration of macrophages hourly over a 6-hour period post-injury. In the control group, macrophage accumulation at the injury site peaked at 3 hours post-injury and remained at a high level through 6 hours. In contrast, sunitinib treatment not only significantly reduced the peak macrophage level but also delayed its occurrence to 4 hours post-injury. Furthermore, macrophage numbers in the sunitinib-treated group were substantially lower than those in the control group throughout the entire 2-to-6-hour observation period (**Figures 3C, D**). Therefore, sunitinib may impair tissue regeneration by inhibiting local macrophage migration, as evidenced by the reduced and delayed macrophage accumulation at the injury site (**Figures 3C, D**).

### 3.4 Transcriptomic analysis identifies PPARγ-related macrophage dysfunction

**Figure 4:**
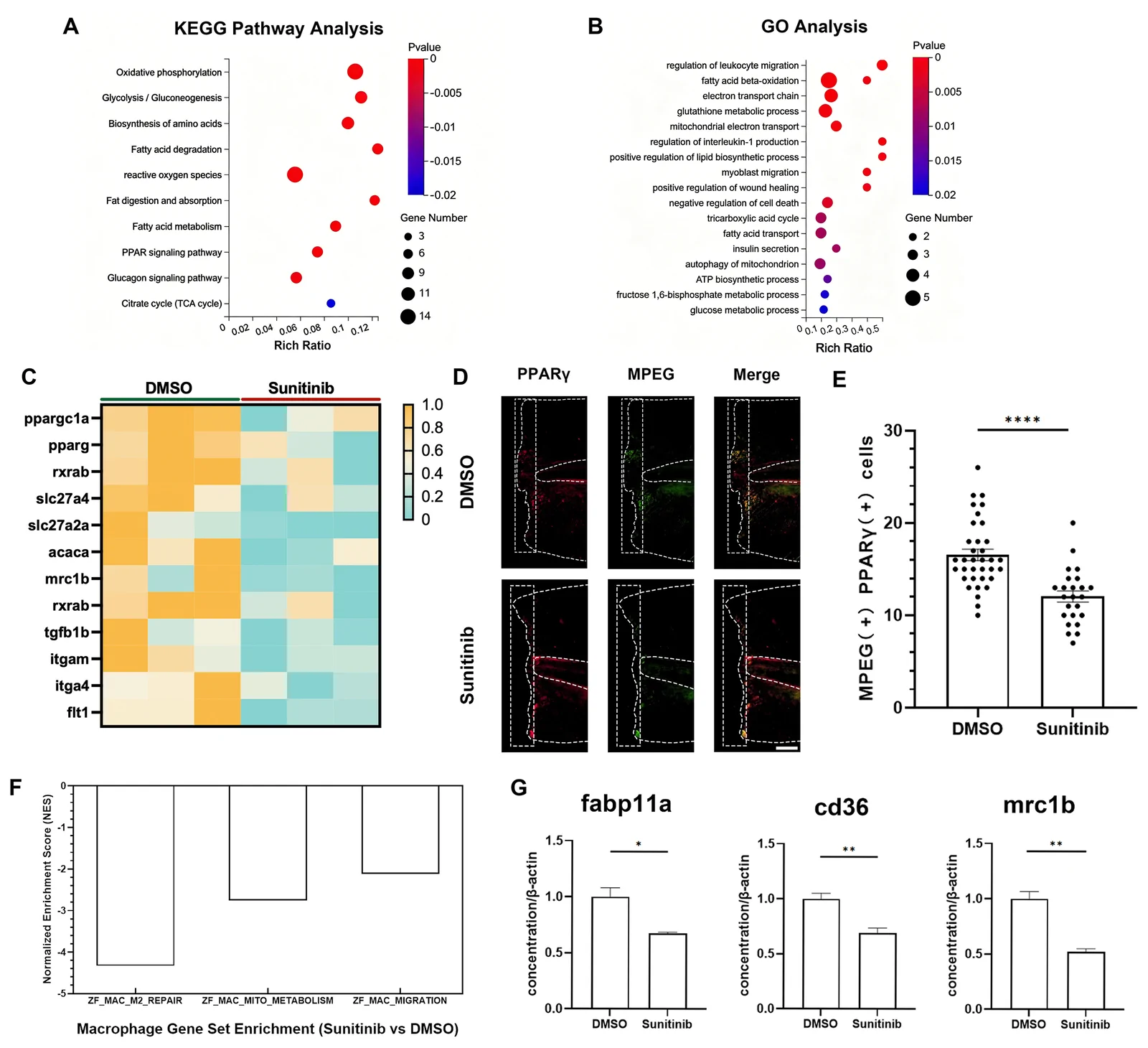
Transcriptomic analysis identifies PPARγ-related macrophage dysfunction. **(A, B)** Zebrafish treated with sunitinib or DMSO were analyzed with transcriptome sequencing. The differentially expressed genes were subjected to functional enrichment analysis including KEGG **(A)** and GO **(B)**. **(C)** Heatmap displaying the expression of genes associated with fatty acid metabolism, PPAR signaling pathway and macrophage function. n=3. **(D)** Representative immunofluorescence images of PPARγ+ macrophages (green) in the tail fin wound region. Macrophages were labeled with GFP antibody (green); PPARγ was detected by antibody (red). Scale bar: 50 μm. **(E)** Quantification of PPARγ+ macrophage density from **(D)**. n=35 in DMSO group, 24 in sunitinib group. **(F)** Gene set enrichment analysis (GSEA) of macrophage-specific transcriptional programs. Normalized enrichment score (NES) for M2/pro-repair signature, mitochondrial/metabolic program, and migration signature. **(G)** RT-qPCR validation of downstream PPARγ targets and reparative macrophage-associated genes. n=3. *p < 0.05, **p < 0.01 and ****p < 0.0001.

To comprehensively characterize the changes induced by sunitinib, we performed transcriptome sequencing with sunitinib treated zebrafish. The results revealed significant alterations in energy metabolism and inflammatory response pathways (**Figures 4A, B**). At the metabolic level, the downregulation of the Oxidative phosphorylation and Citrate cycle pathways indicates a severe impairment in mitochondrial bioenergetics. The enrichment analysis also highlighted significant changes in pathways related to tissue repair and inflammation. Specifically, the regulation of leukocyte migration and positive regulation of wound healing processes were affected, which mechanistically explains the delayed tissue regeneration and macrophage recruitment defects observed in sunitinib-treated phenotypes (**Figures 1**, **3**). Furthermore, the regulation of interleukin-1 production suggests that sunitinib may alter the pro-inflammatory microenvironment, potentially contributing to impaired cellular function. Consistently, genes associated with Fatty acid ​ and the PPAR signaling pathway were also detected (**Figures 4A–C**). PPARγ signaling plays critical roles in macrophages fate determination and phagocytic function, thereby contributing indispensably to immune homeostasis, tissue repair and wound healing (18). The expression of PPARγ was verified with immunofluorescence staining, and the results showed that sinitinib significantly decreased the number of PPARγ+ macrophage in zebrafish (**Figures 4D, E**). Then the gene set enrichment analysis was performed to determine whether macrophage-specific transcriptional programs were altered. The results showed that the M2/pro-repair macrophage signature and the mitochondrial/metabolic macrophage program were downregulated significantly (**Figure 4F**). In addition, although the macrophage migration signature did not reach statistical significance (p = 0.06), the downward trend was consistent with the impaired macrophage recruitment observed in our functional assays (**Figure 3**). Then the mRNA levels of several downstream targets and reparative macrophage-associated genes were verified by RT-qPCR (**Figure 4G**). Therefore, sunitinib may induce macrophage dysfunction via PPARγ-related pathway.

### 3.5 Rosiglitazone alleviated regenerating disorder induced by sunitinib

**Figure 5:**
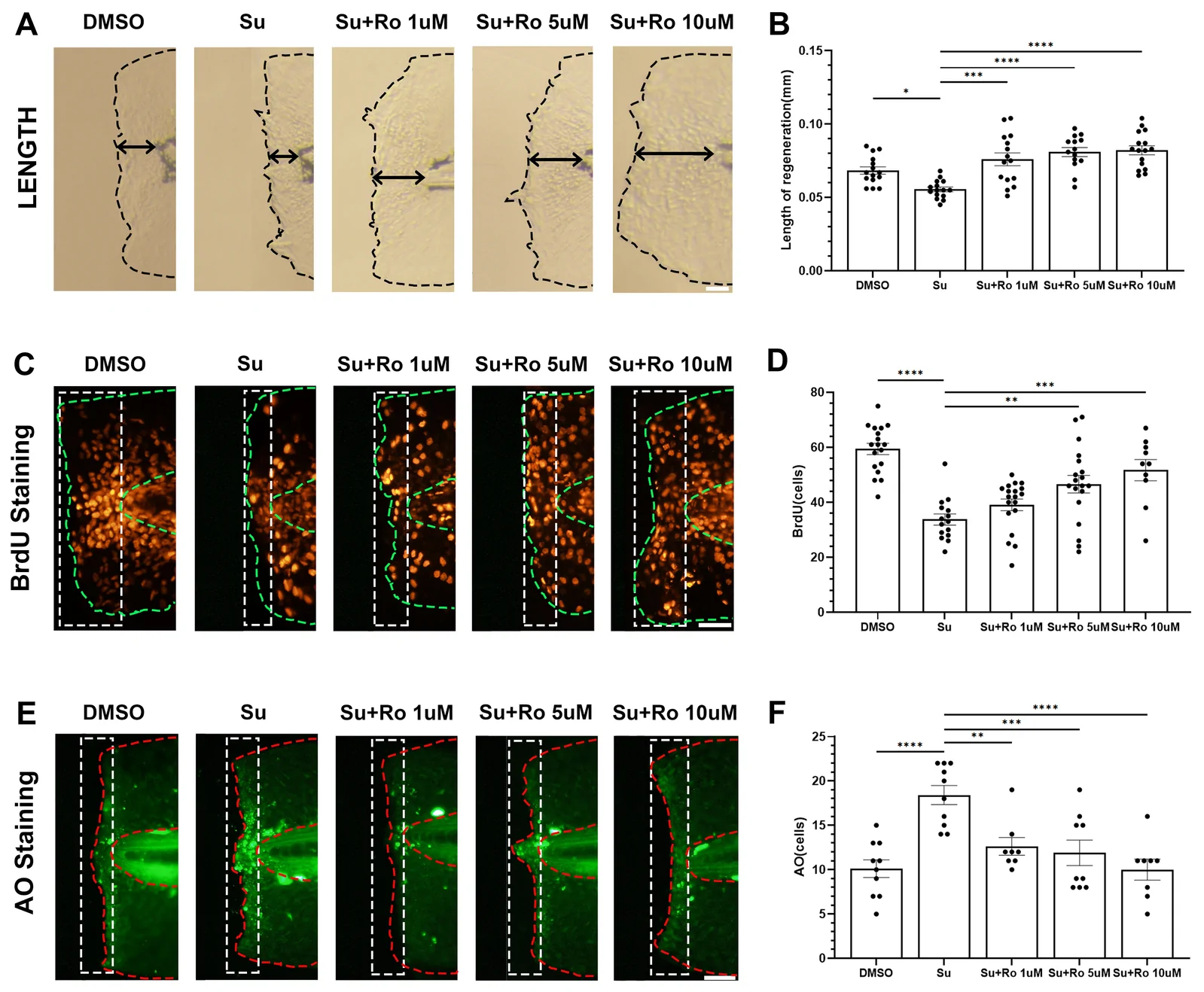
Rosiglitazone alleviated regenerating disorder induced by sunitinib. **(A)** Images of tail fin two days after injury. Black arrow: The distance from the distal end of the notochord at the level of the spinal column to the edge of the regenerating tissue was measured as the regeneration length. Scale bar: 50 μm. **(B)** Quantification of the length data from **(A)**. n=15 in DMSO, Su and Su+Ro 1 mM group, 14 in Su+Ro 5 mM group, 16 in Su+Ro 10 mM group. **(C)** Detection of proliferating cells by fluorescence microscopy one day after tail fin injury. Proliferating cells were labeled with BrdU (orange). Green dashed line: Outline of the zebrafish tail fin and spinal column. White box: Proliferating cells within the quantified region. Scale bar: 50 μm. **(D)** Quantification of the cell count data from **(C)**. n=18 in DMSO group, 15 in Su group, 19 in Su+Ro 1 mM group, 19 in Su+Ro 5 mM group, 10 in Su+Ro 10 mM group. **(E)** Detection of apoptotic cells by fluorescence microscopy six hours after tail fin injury. Apoptotic cells were labeled with AO (green). Red dashed line: Outline of the zebrafish tail fin and spinal column. White box: Apoptotic cells within the quantified region. Scale bar: 50 μm. **(F)** Quantification of the cell count data from **(E)**. n=10 in DMSO and Su group, 8 in Su+Ro 1 mM group, 9 in Su+Ro 5 mM group, 8 in Su+Ro 10 mM group. **p < 0.01, ***p < 0.001, and ****p < 0.0001.

To determine whether PPARγ activation can mitigate or reverse the detrimental effects of sunitinib on tissue repair, the PPARγ agonist rosiglitazone was administrated to the sunitinib-treated zebrafish. The results demonstrated that the rosiglitazone + sunitinib group exhibited significantly increased tail fin regeneration length, rescuing the repair dysfunction induced by sunitinib (**Figures 5A, B**). Consistent findings were observed in the assessment of proliferating and apoptotic cells within the regenerating tail fin tissue (**Figures 5C–F**). Consistent with these findings, rosiglitazone effectively reduced sunitinib-induced tissue apoptosis, enhanced cell proliferation, and restored tissue repair and regeneration.

### 3.6 Rosiglitazone restored the function of macrophages

**Figure 6:**
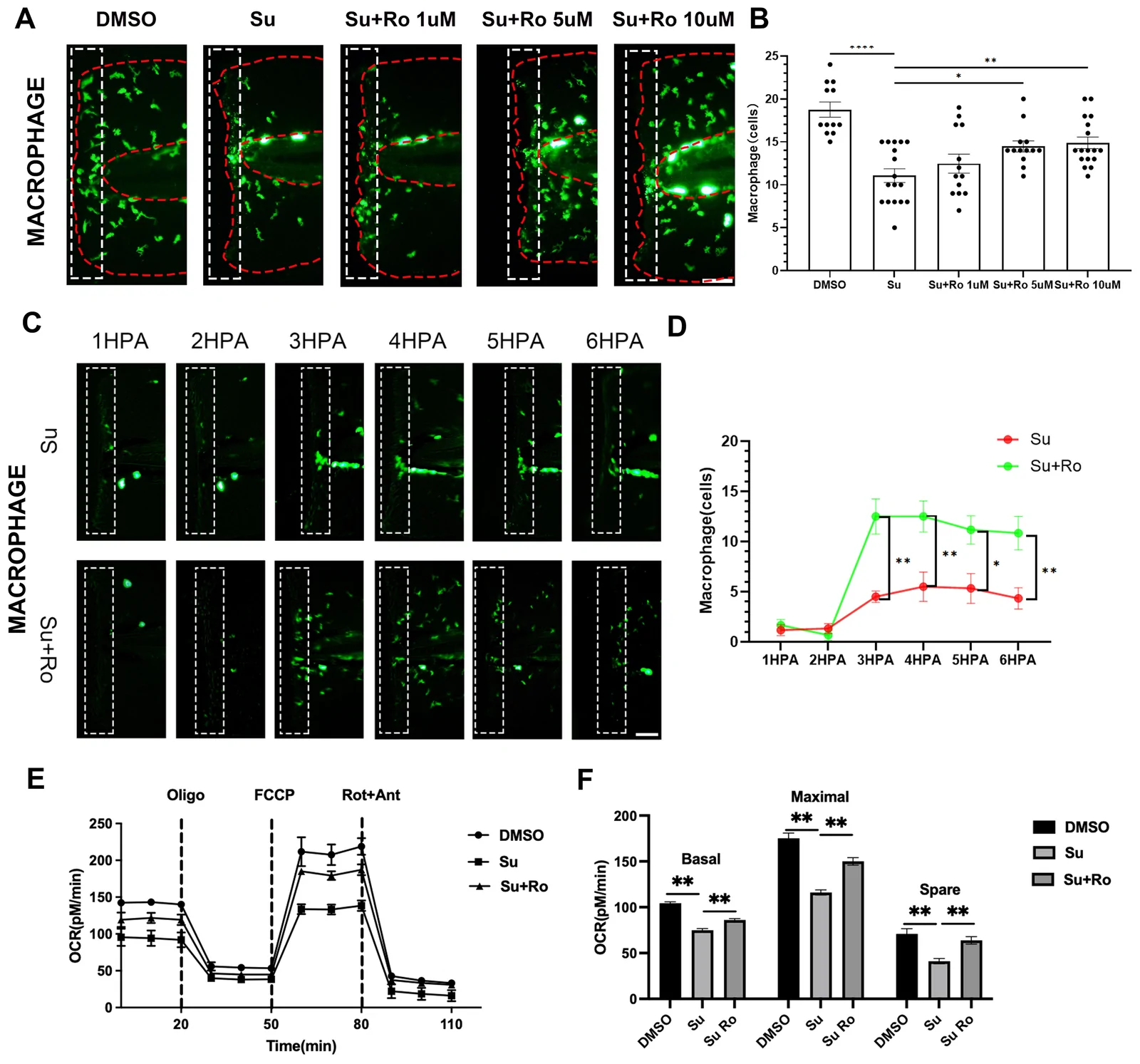
Rosiglitazone restores macrophage function. **(A)** Image of tail fin six hours after injury. Macrophages were labeled with MPEG (green). Red dashed line: Outline of the zebrafish tail fin and spinal column. White box: Macrophages within the quantified region. Scale bar: 50 μm. **(B)** Quantification of the cell count data from **(A)** is shown in **(B)**. n=12 in DMSO, 17 in Su group, 13 in Su+Ro 1μM group, 13 in Su+Ro 5μM group, 17 in Su+Ro 10μM group. **(C)** Images recorded hourly over six hours following tail fin injury. Macrophages were labeled with MPEG (green). White box: Macrophages within the quantified region. Scale bar: 50 μm. **(D)** Quantification of the data from **(C)** is shown in **(D)**. n=6. **(E)** Macrophage from zebrafish were collected and the mitochondrial respiration was assessed using the Seahorse XF mito stress test. **(F)** The basal OCR, maximal respiratory capacity and spare respiratory capacity of macrophage were measured. n=3. *p < 0.05, **p < 0.01, and ****p < 0.0001.

To verify whether rosiglitazone affects macrophages in the regenerating tail fin tissue, the infiltration level of macrophages was examined in Tg (MPEG: GFP) transgenic zebrafish. The results showed that the number of macrophages in the regenerating tail fin tissue was restored after rosiglitazone treatment (**Figures 6A, B**). Dynamic imaging at hourly intervals in the 5 μM rosiglitazone group revealed a significant increase in macrophage numbers after 3 hours (**Figures 6C, D**). The transcriptomic analysis revealed that mitochondrial metabolism was significantly affected by sunitinib (**Figures 4A, B**)). Consistent with our transcriptomic data, sunitinib treatment significantly compromised oxidative phosphorylation (**Figure 6E**). Specifically, sunitinib-treated macrophages exhibited a substantial decrease in basal oxygen consumption rate (OCR). Furthermore, the maximal respiratory capacity and spare respiratory capacity were also significantly diminished, indicating a reduced ability of these cells to respond to energetic stress, a critical requirement for their migration and effector functions during tissue repair (**Figure 6F**). This was accompanied by an enrichment in the Glycolysis/Gluconeogenesis pathway and Glucagon signaling pathway, suggesting a metabolic shift towards aerobic glycolysis to compensate for the reduced ATP production from oxidative breakdown (**Figures 4A, B**). Given that PPAR signaling is a master regulator of fatty acid oxidation and mitochondrial function, its suppression provides a molecular explanation for the observed decrease in fatty acid utilization and mitochondrial respiration. Importantly, this sunitinib-induced suppression of OXPHOS was effectively reversed by co-treatment with the PPARγ agonist rosiglitazone (**Figures 6E, F**). These results demonstrate that sunitinib may inhibits oxidative phosphorylation in macrophages, providing a functional metabolic basis for the observed impairment in macrophage function. The rescue of mitochondrial function by PPARγ activation may restored the ability of macrophages.

### 3.7 Depletion of macrophages abrogates the rescuing effect of rosiglitazone

**Figure 7:**
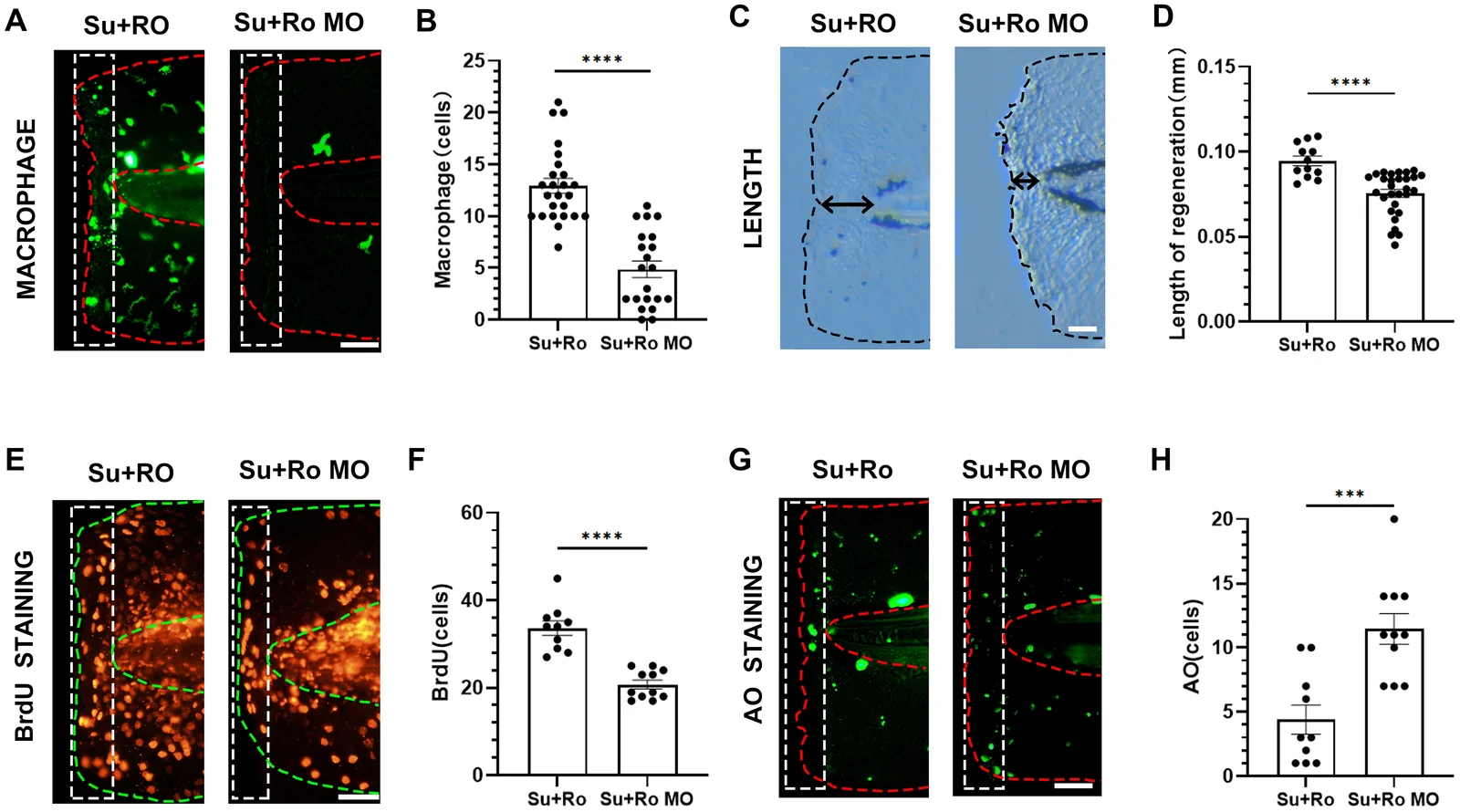
Inhibition of macrophages blocks the rescuing effect of rosiglitazone. **(A)** Image of tail fin six hours after injury. Macrophages were labeled with MPEG (green). Red dashed line: Outline of the zebrafish tail fin and spinal column. White box: Macrophages within the quantified region. Scale bar: 50 μm. **(B)** Quantification of the cell count data from **(A)** is shown in **(B)**. n=25 in Su+Ro group and 21 in Su+Ro MO group. **(C)** Images of tail fin two days after injury. Black arrow: The distance from the distal end of the notochord at the level of the spinal column to the edge of the regenerating tissue was measured as the regeneration length. Scale bar: 50 μm. **(D)** Quantification of the length data from **(C)** is shown in **(D)**. n=12 in Su+Ro group and 29 in Su+Ro MO group. **(E)** Detection of proliferating cells by fluorescence microscopy one day after tail fin injury. Proliferating cells were labeled with BrdU (orange). Green dashed line: Outline of the zebrafish tail fin and spinal column. White box: Proliferating cells within the quantified region. Scale bar: 50 μm. **(F)** Quantification of the cell count data from **(E)** is shown in **(F)**. n=10 in Su+Ro group and 11 in Su+Ro MO group. **(G)** Detection of apoptotic cells by fluorescence microscopy six hours after tail fin injury. Apoptotic cells were labeled with AO (green). Red dashed line: Outline of the zebrafish tail fin and spinal column. White box: Apoptotic cells within the quantified region. Scale bar: 50 μm. **(H)** Quantification of the data from **(G)** is shown in **(H)**. n=10 in Su+Ro group and 11 in Su+Ro MO group. ***p < 0.001, and ****p < 0.0001.

To verify whether rosiglitazone rescues sunitinib-impaired tail fin repair by restoring macrophage function, we performed macrophage knockdown via MO technology in sunitinib- and rosiglitazone-treated zebrafish. The results showed that the number of macrophages in the regenerating tail fin tissue was reduced (**Figures 7A, B**), leading to decreased tail fin regeneration length (**Figures 7C, D**), fewer proliferating cells (**Figures 7E, F**), and increased apoptotic cells (**Figures 7G, H**) within the tissue. The rescuing effect of rosiglitazone was attenuated, and tissue regeneration was again suppressed. These findings demonstrated that rosiglitazone may its therapeutic role by modulating macrophage function.

## 4 Discussion

Our findings indicate that the anti-angiogenic agent sunitinib suppresses the PPARγ signaling pathway and impedes tissue regeneration, an effect that may be associated with macrophage dysfunction. Importantly, the PPARγ agonist rosiglitazone reverses this process, an effect dependent on the presence of macrophages. This work not only reveals a novel mechanism underlying the side effects of sunitinib but also proposes a potential therapeutic strategy for drug-induced healing disorder, particularly associated with anti-angiogenic agents. Sunitinib is a multi-targeted tyrosine kinase inhibitor with effects on endothelial cell, fibroblast, epithelial and multiple biological processes (3, 5, 19). While our study found that macrophage dysfunction might be a key contributor to impaired regeneration, we cannot exclude the possibility that the inhibition of angiogenesis, endothelial cell dysfunction, altered fibroblast behavior, suppressed epithelial proliferation, and impaired vascular remodeling compromise tissue regenerative capacity. Thus, the regenerative deficit observed in sunitinib-treated animals likely reflects combined effects across multiple cell types, rather than being exclusively driven by macrophage dysfunction. Future work employing cell-type-specific approaches will be needed to disentangle these overlapping mechanisms.

PPARγ, a member of the nuclear receptor superfamily, serves as a key transcriptional regulator of lipid metabolism, inflammation, and reparative macrophage-associated genes. Our transcriptomic sequencing analysis revealed a significant suppression of this pathway following sunitinib treatment. Previous studies have well-established that PPARγ modulates multiple core functions of macrophages including tissue repair, metabolic reprogramming, efferocytosis, migration, and recruitment. Thus, PPARγ plays a central role in maintaining homeostasis, regulating inflammatory responses, and promoting tissue repair. For example, PPARγ acts as a key molecular driving macrophage towards the alternatively activated/repair phenotype by transactivating a set of M2-associated genes (e.g., Arg1, Mrc1, IL-10) while simultaneously suppressing pro-inflammatory signaling pathways such as NF-κB, thereby inhibiting pro-inflammatory phenotype (20). Furthermore, PPARγ promotes mitochondrial biogenesis and fatty acid oxidation, providing the necessary energy and biosynthetic precursors for M2 macrophages to perform sustained repair functions (21). PPARγ also regulates macrophage migratory behavior, with effects that can be microenvironment-dependent (22). In tissue repair process, PPARγ may facilitate the appropriate localization and retention of macrophages within tissues, potentially by regulating integrins and cytoskeleton-associated proteins, thereby ensuring their repair functions. This effect may explain the observed reduction in macrophage infiltration upon PPARγ signaling inhibition in our study. As mentioned above, macrophages exhibit diverse phenotypes for its different functions. While the current study investigated the impact of sunitinib on altered expression of reparative macrophage-associated genes and infiltration levels, it did not explore the definitive macrophage polarization. This intriguing aspect remains to be further explored.

Rosiglitazone, a classic PPARγ agonist, is used for type 2 diabetes mellitus. Recent studies have revealed that PPARγ agonists play significant roles in regulating immune responses, reparative macrophage polarization, and enhancing cellular metabolism in acute kidney injury, ulcerative colitis (23, 24), as well as in airway disease and atherosclerosis (25, 26). In this study, rosiglitazone was used to counteract the inhibition of PPARγ, which successfully increased local macrophage infiltration and improved wound healing. This approach provides a clinically validated and safe therapeutic option for sunitinib-induced tissue repair disorder, holding significant clinical relevance. However, it should be noted that rosiglitazone is known to act on multiple cell types including endothelial cells, fibroblasts, and epithelial cells, as well as systemic metabolism (27–29). Therefore, contributions from non-macrophage targets or indirect systemic effects cannot be ruled out. Future studies employing macrophage-specific PPARγ knockout models or selective PPARγ agonists with restricted cellular distribution would be required to definitively attribute the regenerative benefit to macrophage-intrinsic PPARγ activation. Additionally, several issues must be addressed before clinical translation. For example, the known cardiovascular risks of rosiglitazone raise safety concerns in cancer patients who may already have compromised cardiac function (30), and whether rosiglitazone interferes with sunitinib’s anti-tumor efficacy remains unknown, as PPARγ activation could theoretically alter tumor microenvironments or drug metabolism.

The zebrafish model, owing to its high fecundity, short life cycle, and high genetic and metabolic homology with mammals, has been widely used in research (31). The transparency of zebrafish embryos and the availability of transgenic lines allowing dynamic tracking of specific cells *in vivo* provide significant advantages for live fluorescence imaging. Well-established zebrafish injury models, including those for the liver, heart, and tail fin, are routinely employed in tissue regeneration research to investigate related cellular and molecular mechanisms (32). However, the zebrafish model has limitations. Although tail fin regeneration in zebrafish is an excellent model for wound healing, the regenerative process is complete, which differs from the limited regenerative capacity and scar formation in humans (33). Consequently, the repair processes observed in zebrafish models may only represent certain aspects of wound healing and may not fully recapitulate the complex pathological process. Studies using clinically relevant MRONJ animal models or patient-derived samples/cells are essential to evaluate the translational potential and clinically achievable plasma concentrations of rosiglitazone.

## 5 Conclusion

This study demonstrates that sunitinib suppresses PPARγ signaling and disrupts macrophage mitochondrial metabolism and migration in a zebrafish regeneration model. Transcriptomic and immunofluorescence analyses confirmed PPARγ pathway inhibition at both the transcriptional and protein levels, along with downregulation of reparative gene programs. Activation of PPARγ with rosiglitazone restored macrophage infiltration, mitochondrial oxidative phosphorylation, and tissue regeneration, whereas macrophage depletion abrogated this rescue. These findings identify PPARγ as a key node in sunitinib-induced macrophage dysfunction and suggest that targeting PPARγ may represent a promising strategy for mitigating sunitinib-associated tissue repair deficits. Further studies using macrophage-specific genetic approaches are warranted to establish direct causality and evaluate translational potential in mammalian systems.

## Data Availability

The data presented in the study are deposited in the Gene Expression Omnibus (GEO) repository, accession number GSE342695.
